# Maternal Hyperglycemia Disrupts Histone 3 Lysine 36 Trimethylation of the IGF-1 Gene

**DOI:** 10.1155/2012/930364

**Published:** 2012-04-04

**Authors:** Erin K. Zinkhan, Qi Fu, Yan Wang, Xing Yu, Christopher W. Callaway, Jeffrey L. Segar, Thomas D. Scholz, Robert A. McKnight, Lisa Joss-Moore, Robert H. Lane

**Affiliations:** ^1^Division of Neonatology, Department of Pediatrics, University of Utah, 295 Chipeta Way, Salt Lake City, UT 84108, USA; ^2^Department of Pediatrics, University of Iowa Hospitals and Clinics, 200 Hawkins Drive, Iowa City, IA 52242, USA

## Abstract

*In utero* environmental adaptation may predispose to lifelong morbidity. Organisms fine-tune gene expression to achieve environmental adaptation by epigenetic alterations of histone markers of gene accessibility. One example of epigenetics is how uteroplacental insufficiency-induced intrauterine growth restriction (IUGR), which predisposes to adult onset insulin resistance, decreases postnatal IGF-1 mRNA variants and the gene elongation mark histone 3 trimethylation of lysine 36 of the IGF-1 gene (H3Me3K36). Limitations in the study of epigenetics exist due to lack of a primary transgenic epigenetic model. Therefore we examined the epigenetic profile of insulin-like growth factor 1 (IGF-1) in a well-characterized rat model of maternal hyperglycemia to determine if the epigenetic profile of IGF-1 is conserved in disparate models of *in utero* adaptation. We hypothesized that maternal hyperglycemia would increase IGF-1 mRNA variants and H3Me3K36. However maternal hyperglycemia decreased hepatic IGF-1 mRNA variants and H3Me3K36. This finding is intriguing given that despite different prenatal insults and growth, both maternal hyperglycemia and IUGR predispose to adult onset insulin resistance. We speculate that H3Me3K36 of the IGF-1 gene is sensitive to the glucose level of the prenatal environment, with resultant alteration of IGF-1 mRNA expression and ultimately vulnerability to adult onset insulin resistance.

## 1. Introduction

An adverse *in utero* nutritional environment predisposes to lifelong morbidity [1]. One way an organism can adapt to its adverse *in utero *nutritional environment is through fine-tuning gene expression. The modification through which reprogramming of gene expression occurs is called epigenetics. The epigenetic profile consists of a series of DNA and histone modifications that allow increased or decreased access of transcription machinery to DNA for fine-tuning of gene expression. Fine-tuning of gene expression and environmental adaptation can occur through alternate promoter or exon usage.

**Figure 1:**
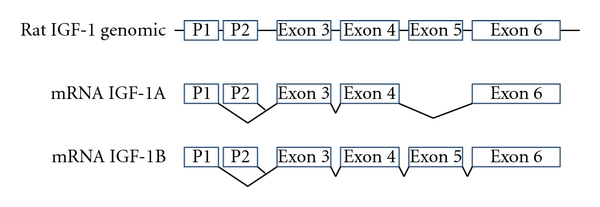
Genomic and mRNA variants of the rat IGF-1 gene. Exons are shown as boxes and introns as lines. IGF-1A lacks exon 5, while IGF-1B contains exon 5. Transcription of IGF-1A and IGF-1B may begin from within either of promoter 1 (P1) or promoter 2 (P2).

IGF-1 exemplifies a classic gene that undergoes epigenetic regulation [2, 3]. IGF-1 is a gene whose protein is responsible for postnatal growth and is involved in insulin sensitivity and can be transcribed from one of two promoters and with alternative splicing of exon 5 (Figure 1). Regulation of IGF-1 variant expression is in part due to alteration of the pattern of DNA methylation and histone modifications around the gene and is sensitive to the perinatal environment [3–5].

Hepatic IGF-1 mRNA levels largely impact serum IGF-1 levels and are heavily modulated by epigenetics in the setting of intrauterine growth restriction (IUGR) [3, 6]. IUGR causes fetal hypoglycemia, decreases postnatal growth, and predisposes to adult onset insulin resistance, both of which are modulated by IGF-1. Observation of the epigenetic profile of the IGF-1 gene from promoter 1 through the 3′ untranslated region (UTR) at day of life (DOL) 21, prior to the onset of insulin resistance, revealed persistent decrease in histone 3 trimethylation of lysine 36 (H3Me3K36) [3], a histone mark that is associated with gene elongation [7–9]. Although the effects of IUGR on the epigenetic profile of the IGF-1 gene and H3Me3K36 in particular are known, further study of the epigenetic profile of the IGF-1 gene has been limited due to lack of a primary transgenic epigenetic model. Therefore we sought an alternative method to study the epigenetic profile of IGF-1, using a disparate model to IUGR to determine whether the vulnerability of H3Me3K36 to perinatal insults is conserved.

The model we chose to study epigenetic profile of the IGF-1 gene is a well-characterized model of streptozotocin- (STZ-) induced maternal hyperglycemia [10]. Maternal hyperglycemia was chosen because the *in utero* effects of maternal hyperglycemia on the fetus are quite different from those seen in the setting of IUGR. In humans, maternal hyperglycemia often generates large for gestational age infants and increases IGF-1 serum levels at birth [11–13], yet it still predisposes to alterations in postnatal growth and to adult onset insulin resistance. In this rat model of maternal hyperglycemia, offspring of hyperglycemic mothers (OHMs) have the same average birth weight but with greater numbers of larger and smaller pups than offspring from control (CON) mothers, OHM males gain less weight after 2 months of age, and OHM males develop insulin resistance by 6 months of age [10]. DOL 21 was chosen for our epigenetic profile evaluation of the IGF-1 gene because it precedes the confounders of adolescence, weight gain, and insulin resistance.

We hypothesized that maternal hyperglycemia in rats would increase offspring serum IGF-1 levels, hepatic IGF-1 mRNA variants, and epigenetic markers of IGF-1 associated with gene elongation. Specifically, rat maternal hyperglycemia would decrease offspring promoter DNA methylation and alter multiple markers of the histone code from promoter 1 through the 3′UTR of the IGF-1 gene including increased H3Me3K36. Further, because of their increased risk of development of insulin resistance in adulthood, we hypothesized that these alterations of IGF-1 will be worse in males.

## 2. Materials and Methods

### 2.1. Animals

Frozen rat serum and liver were generously given by Dr. Segar at the University of Iowa. Animals from different litters were used for each experiment with an *n* = 3–6 per sex and per treatment for a total of 24 animals for serum glucose and IGF-1 experiments and a total of 15 animals for liver mRNA and epigenetic profile experiments. Animal procedures were previously described [10]. In brief, there was no difference in litter size between OHM and CON [10]. Offspring birth weights did not differ between OHM and CON, though with greater variability among OHM such that there were more large and small pups. Single serum glucose was determined at necropsy using the LifeScan One Touch Ultra Blood Glucose Monitoring System (LifeScan Inc, Milpitas, CA), for a total of *n* = 6 per sex per treatment.

### 2.2. Enzyme Immunoassay

Serum from DOL 21 OHM was generously given by Dr. Segar at the University of Iowa. Serum IGF-1 levels at DOL 21 were measured in triplicate with the Quantikine Mouse/Rat IGF-1 enzyme immunoassay kit following the manufacturer's protocol (R&D Systems, Inc., Minneapolis, MN, USA).

### 2.3. RNA Isolation

Total RNA was isolated from frozen liver at DOL 21 as previously described [14] using the Nucleospin RNAII kit (Machery-Nagel, Bethlehem, PA, USA), including DNase I treatment. RNA was quantitated with a spectrophotometer and checked by gel electrophoresis for integrity. The cDNA was synthesized from 1 *μ*g RNA using the High-Capacity cDNA Reverse Transcription Kit (Applied Biosystems, Foster City, CA, USA) per manufacturers' protocol.

### 2.4. Real-Time Reverse Transcriptase Polymerase Chain Reaction (RT-PCR)

Real-time RT-PCR was performed as described previously [14]. Primers and probes were the same as used previously [3]. Semiquantitative real-time RT PCR quantification was then performed using glyceraldehydes-3 phosphate dehydrogenase (GAPDH) as an internal control, as Ct values of GAPDH did not differ between CON and OHM animals [15]. Relative quantification of PCR products was based on differences between GAPDH and the target using the comparative Ct method (TaqMan Gold RT-PCR manual; PE Biosystems, Foster City, CA, USA).

### 2.5. Chromatin Immunoprecipitation (ChIP) Assay and Real-Time PCR

ChIP with 1 *μ*L anti-H3AcK14, anti-H3Me2K4, anti-H3Me3K4 (Millipore Upstate, Charlottesville, VA, USA), and anti-H3Me3K36 (Abcam, Cambridge, MA, USA) was performed as described previously for chromatin from DOL 21 livers [3, 14]. Chromatin equivalent to 100 *μ*g DNA based on A260 absorption was used in each immunoprecipitation (IP) reaction. After purification from IP chromatin, DNA was determined with a standard curve with SYBR Safe DNA gel stain (Molecular Probes, Eugene, OR, USA). The SYBR Safe fluorescence was measured using a Tecan plate reader (Genios Pro-Basic w/o FP; Tecan Austria GmbH, Grodig, Austria) and Magellan V 6.2 software (Tecan). DNA fragments containing IGF-1 site-specific sequences including the seven regions of interest (P1, P2, exon 3, exon 4, exon 5, and proximal and distal 3′untranslated region (UTR) of exon 6) of the IGF- 1 gene were quantified by real-time PCR [3]. Primer and probe sequences are the same as described previously [3], and for exon 3 the following primers were used: forward primer AGACGGGCATTGTGGATGA, reverse primer TCCTGGGTGTGCCTTTGAC, and probe TGTTGCTTCCGGAGCT. Relative quantification of PCR products was based on value differences between the target and intergenic control using the comparative Ct method [15]. An intergenic region upstream of the IGF-1 gene was chosen as a well-characterized nontranscribing region of DNA that contains the histone marks of interest in this series of experiments as described by Fu et al. [3]. The distribution pattern of histone modifications along IGF-1 was determined by looking at the seven sites as indicated previously and expressed as a percentage of the values obtained for P1.

### 2.6. Bisulfite Modification

Bisulfite modification was performed as described previously using the primers as described previously [3, 14]. PCR conditions were 95°C for 10 minutes, then 94°C for 30 seconds, annealing at either 53°C or 54°C for 30 seconds depending on the primers, and 72°C for 30 seconds, for 35 cycles. PCR products were cloned into the vector for pSC-A (Stratagene, Cedar Creek, TX, USA). Six to eight colonies from each PCR cloning reaction were inoculated into SeqPrep 96 well plates (Edge BioSystems, Gaithersburg, MD, USA). The plasmid DNA was prepared using the SeqPrep 96 Plasmid Prep Kid (Edge BioSystems) and sequenced according to protocol using the BigDye Terminator v3.1 Cycle Sequencing kit (Applied Biosystems) with M13 forward or reverse primers.

### 2.7. Statistics

Data were presented as a mean ± standard error (SE) percent of control or of P1. ANOVA (Fisher's protected least-significant difference) and Student's unpaired *t*-test were used for real-time RT PCR. Student's 2-tailed *t*-test was used for DNA methylation. A value of *P* < 0.05 was considered to be statistically significant.

## 3. Results

### 3.1. Hepatic IGF-1 mRNA and Serum Levels

**Figure 2:**
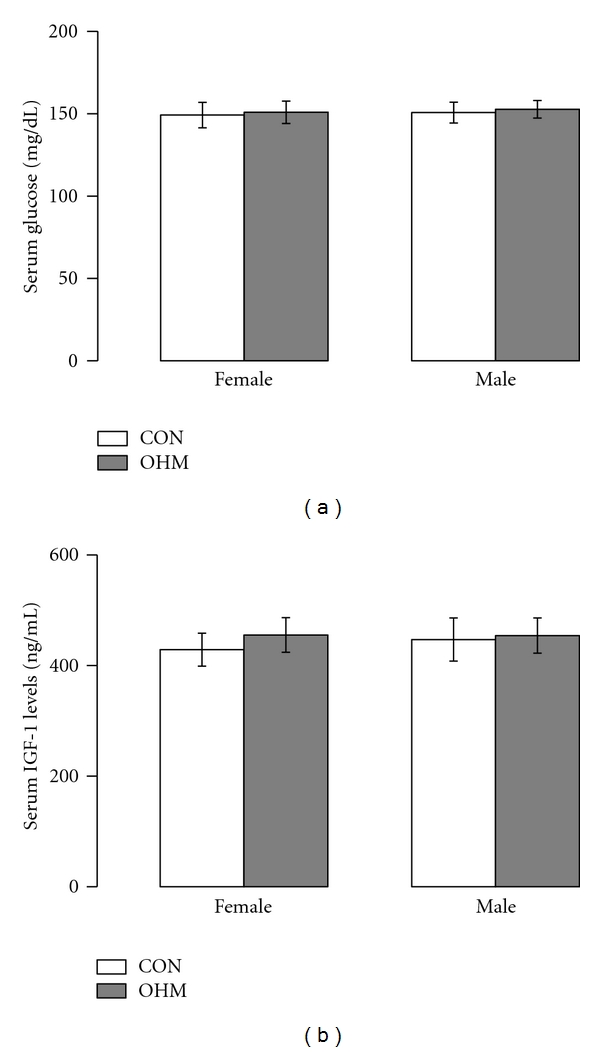
Maternal hyperglycemia did not decrease offspring glucose and serum IGF-1 levels. (a) DOL 21 OHM glucose levels for female and male offspring, CON in white bars and OHM in grey bars. (b) DOL 21 OHM serum IGF-1 levels for female and male offspring, CON in white bars and OHM in grey bars. Serum glucose and IGF-1 levels were expressed as mean ± standard error of the mean, *n* = 6 CON female, *n* = 6 OHM female, *n* = 6 CON male, and *n* = 6 OHM male.

**Figure 3:**
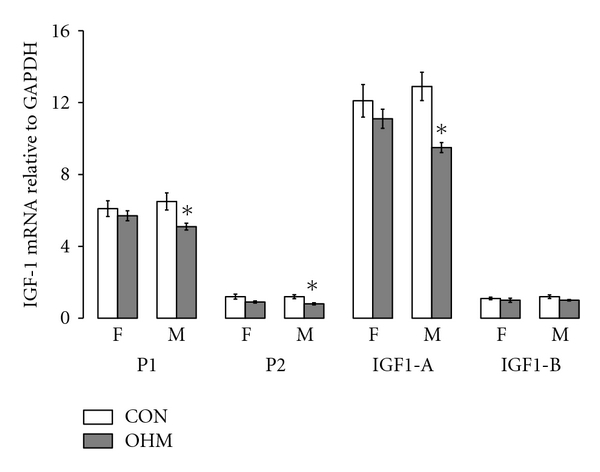
Maternal hyperglycemia decreased hepatic IGF-1 mRNA variants in males. DOL 21 OHM hepatic mRNA variants were expressed as mean ± standard error of the mean relative to GAPDH, for female (F) and male (M) offspring, CON in white bars and OHM in grey bars, *n* = 4 CON female, *n* = 3 OHM female, *n* = 5 CON male, and  *n* = 3 OHM male. *P < 0.05.

No difference in serum glucose and IGF-1 levels was detected between OHM and CON at DOL 21 (Figure 2). Maternal hyperglycemia significantly decreased male promoter 1 (P1), promoter 2 (P2), and IGF-1A mRNA levels (Figure 3). No changes were seen in female IGF-1 mRNA levels (Figure 3).

### 3.2. Hepatic IGF-1 Histone Code

Seven sites along the hepatic IGF-1 gene were analyzed for four histone H3 covalent modifications in the control group to determine a normal histone code along the hepatic rat IGF-1 gene. These same seven sites were analyzed in OHM to determine the effect of maternal hyperglycemia on these sites. Each modification in both CON and OHM groups was normalized to the intergenic region. Each modification was then expressed as a percent of P1.

**Figure 4:**
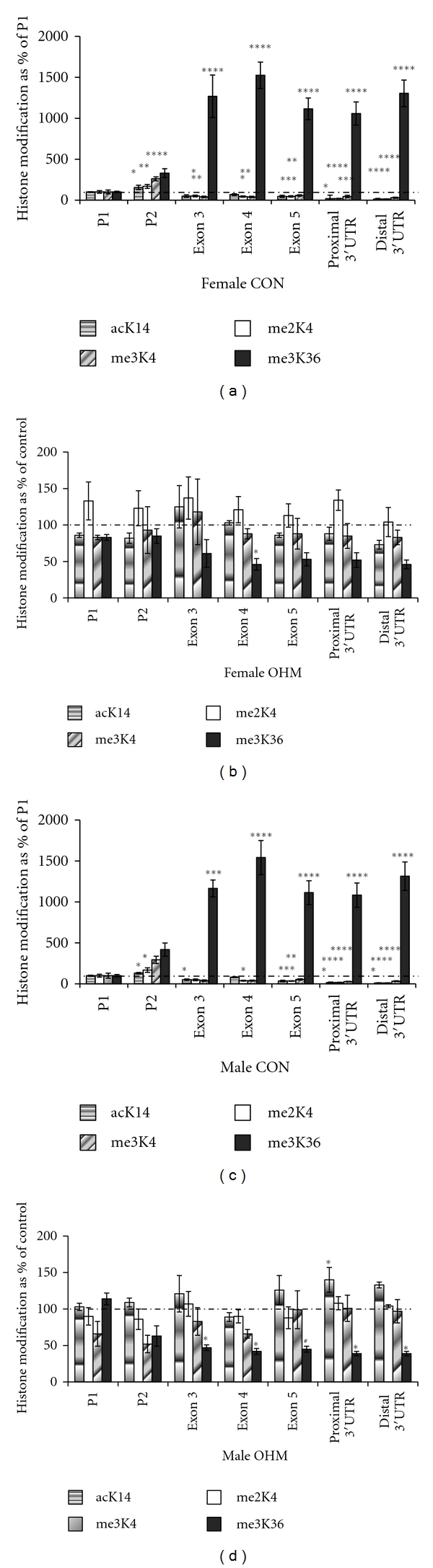
Maternal hyperglycemia decreased H3Me3K36 along the length of the IGF-1 gene in males. (a) Female IGF-1 histone modifications in control animals expressed as a mean percent of P1 ± standard error of the mean. (b) Female IGF-1 histone modifications in OHM animals expressed as a mean percent of gender matched control values ± standard error of the mean. (c) Male IGF-1 histone modifications in control animals expressed as a mean percent of P1 ± standard error of the mean. (d) Male IGF-1 histone modifications in OHM animals expressed as a mean percent of gender matched control values ± standard error of the mean, *n* = 4 CON female, *n* = 3 OHM female, *n* = 5 CON male, and *n* = 3 OHM male. *P < 0.05; **P < 0.01; ***P < 0.001; ****P < 0.0001.

At DOL 21, acetylation at lysine 14 is increased at P2 and then decreased along the rest of the IGF-1 gene relative to P1 in both genders. Similarly, di- and trimethylation are increased at P2 and decreased along the remainder of the IGF-1 gene in both genders. Trimethylation of lysine 36 was increased at all sites relative to P1 in both genders (Figure 4).

These seven sites were also analyzed in the offspring of hyperglycemic dams. Results were presented relative to controls where controls were considered to be 100%. At DOL 21, maternal hyperglycemia increased acetylation of lysine 14 in males distally, with no change seen in females. No change was seen in either di- or trimethylation of lysine 4 in either gender. Maternal hyperglycemia decreased trimethylation of lysine 36 throughout the IGF-1 gene in males, and at exon 4 in females (Figure 4).

### 3.3. IGF-1 P1 Methylation

**Figure 5:**
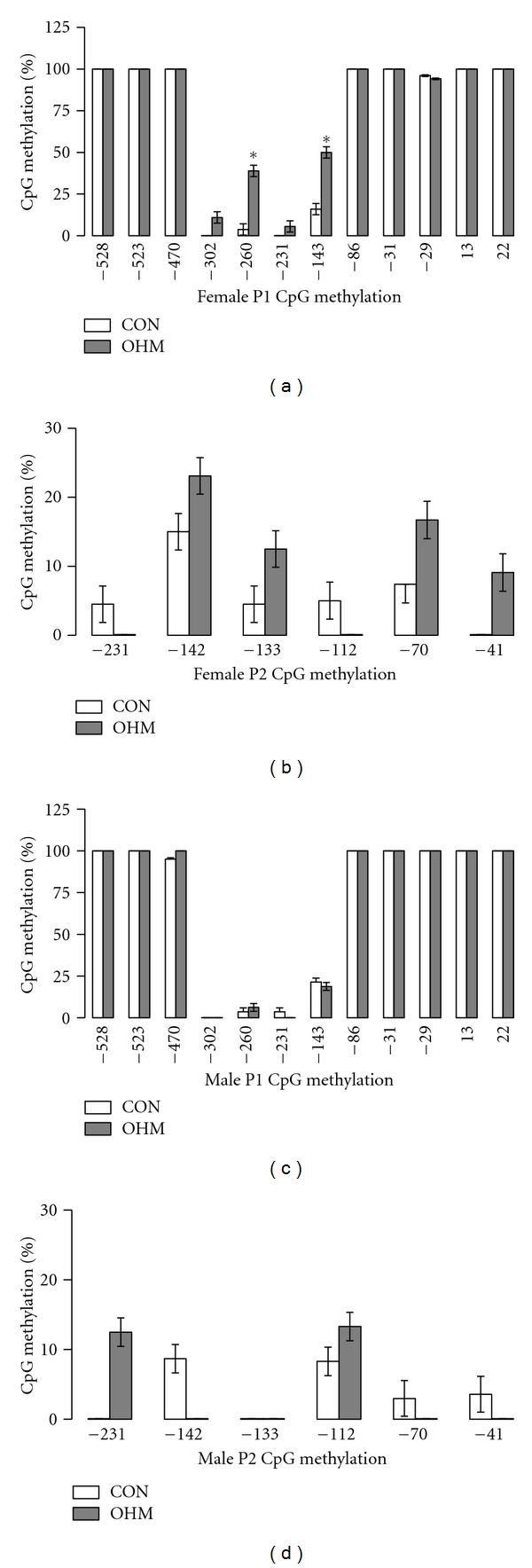
Maternal hyperglycemia increased rat promoter 1 CpG methylation in females. (a) Rat promoter 1 CpG methylation in females. (b) Rat promoter 2 CpG methylation in females. (c) Rat promoter 1 CpG methylation in males. (d) Rat promoter 2 CpG methylation in males. All data were expressed as mean percent of methylation ± standard error of the mean, *n* = 4 CON female, *n* = 3 OHM female, *n* = 5 CON male, and *n* = 3 OHM male. *P < 0.05.

Twelve CpG sites within P1 of IGF-1 were analyzed for methylation. Maternal hyperglycemia significantly increased methylation in females at sites −260 and −143. No difference was seen in methylation at P1 in males (Figure 5).

### 3.4. IGF-1 P2 Methylation

Six CpG sites within P2 of IGF-1 were analyzed for methylation. No significant differences were seen in either gender (Figure 5).

## 4. Discussion

The most important finding of this study is that maternal hyperglycemia decreased H3Me3K36 of the IGF-1 gene in the same fashion that IUGR decreased this histone mark [3]. This finding is most intriguing given that both maternal hyperglycemia and IUGR have the same phenotype of adult onset insulin resistance. We speculate that H3Me3K36 of the IGF-1 gene is sensitive to the glucose level of the prenatal environment, with resultant alteration of IGF-1 mRNA expression and ultimately vulnerability to adult onset insulin resistance.

Maternal hyperglycemia decreased hepatic H3Me3K36 of the IGF-1 gene in DOL 21 OHM males. This histone mark is often associated with mRNA elongation and actively transcribed genes [7–9]. The protein that places the H3Me3K36 mark, one example of which is Wolf-Hirschhorn Syndrome Candidate 1, associates itself with RNA polymerase 2, the enzyme responsible for mRNA transcription, and thus influences mRNA levels [9, 16]. H3Me3K36 appears to be particularly sensitive to alteration by glucose given the similarity of findings between our study of OHM and of IUGR [3]. Decreased H3Me3K36 of the IGF-1 gene occurred in both genders in our prior study of IUGR, and these findings were associated with decreased IGF-1 mRNA variant levels and adult onset insulin resistance in both genders [3, 17].

H3Me3K36 decreases in the setting of either increased or decreased glucose [3]. One possible explanation is that H3Me3K36 is not sensitive to glucose levels themselves but rather other alterations seen in the setting of both maternal hyperglycemia and IUGR, including acidosis and hypoxia. Maternal hyperglycemia leads to fetal acidosis and hypoxia resulting from the increased metabolic activity associated with excess nutrient delivery [18]. IUGR leads to fetal acidosis and hypoxia through decreased nutrient and oxygen delivery from UPI.

Decreased H3Me3K36 of the IGF-1 gene is consistent with the finding of decreased IGF-1 mRNA variant levels in males. Large males from hyperglycemic dams subsequently do not gain weight as quickly and have lower glucose levels by 2 months of age compared to their control counterparts [10], a finding which is *preceded* by decreased IGF-1 mRNA levels in male offspring. This finding suggests decreased male IGF-1 mRNA levels as one mechanism through which large male offspring develop insulin resistance and postnatal growth restriction.

Of note, although maternal hyperglycemia decreases hepatic H3Me3K36 in males, few gender differences were seen in the CON offspring. For example, histone acetylation, also a marker for gene activation [19], of IGF-1 P2 is increased relative to P1 beyond the neonatal period in both genders, a finding seen in other studies [3, 20]. Increased P2 histone acetylation beyond the neonatal period may be an effect of growth hormone signaling [21, 22]. The similarity in histone acetylation and methylation markers and mRNA variant levels occurred in control animals despite housing the animals in different locations and utilizing different control interventions. Similarities that exist in the control animal histone code along IGF-1 reinforced the normal pattern of H3 acetylation and methylation along the IGF-1 gene.

DNA methylation of IGF-1 promoters 1 and 2 remains unchanged in males and is increased in females in this study. DNA methylation is a factor in driving histone code changes [4, 5]. DNA methylation may also aid in nucleosome positioning and thereby effect mRNA transcription [5]. Nucleosomes are approximately 150 base pairs in length, and we found an approximately 150 base pair region of P1 that had less methylation than either 5′ or 3′ of this region in both genders. Thus the increase in DNA methylation in female DOL 21 OHM may contribute to decreased female IGF-1 mRNA variant levels that did not reach statistical significance.

Maternal hyperglycemia did not change serum IGF-1 levels in DOL 21 OHM. IGF-1 serum levels are still present in the setting of decreased or absent hepatic IGF-1 mRNA production, such as the IGF-1 mouse knockout model [2]. The ability of the IGF-1 knockout to maintain serum IGF-1 levels can be achieved through a variety of IGF-1 binding proteins which hold IGF-1 within the serum and may contribute to the normal serum IGF-1 levels in our study [2]. Further, IGF-1 often acts through a paracrine fashion, and thus the importance of decreased IGF-1 mRNA variant levels is in the ability to fine tune IGF-1 expression and may not be reflected completely in serum IGF-1 levels.

One limitation of this study is that maternal hyperglycemia was induced by STZ, and it is possible that STZ crosses the placenta and thus affects fetal and postnatal glucose levels, hepatic IGF-1 mRNA variant levels, and epigenetic characteristics. However, maternal hyperglycemia did not increase glucose levels at DOL 21, indicating that few, if any, offspring pancreatic beta cells were affected directly by STZ. It is also unlikely that STZ directly affected hepatic IGF-1 mRNA variant levels and epigenetic characteristics because STZ is not known to effect the hepatocyte. A further limitation is the small *n* used in this study. However, despite the small *n* we were able to see significant changes in the epigenetic profile of IGF-1 and are consistent with results seen by Fu et al. in the setting of IUGR [3].

In conclusion, maternal hyperglycemia decreases DOL 21 male rat offspring hepatic IGF-1 mRNA variant levels and H3Me3K36 of the IGF-1 gene. These findings are most intriguing given that IUGR also decreases hepatic IGF-1 mRNA variant levels and H3Me3K36 of the IGF-1 gene, and both maternal hyperglycemia and IUGR increase the risk of the same outcome of adult onset insulin resistance. We speculate that decreased H3Me3K36 of the IGF-1 gene in both maternal hyperglycemia and IUGR leads to decreased IGF-1 mRNA variant levels and contributes to the development of adult onset insulin resistance.

## Abbreviations

IGF-1: Insulin like growth factor 1
IUGR: Intrauterine growth restriction
OHM: Offspring of hyperglycemic mothers
DOL: Day of life
ChIP: Chromatin immunoprecipitation
H3Me3K36: Histone 3 trimethylation of lysine 36
H3AcK14: Histone 3 acetylation of lysine 14
H3Me2K4: Histone 3 dimethylation of lysine 4
H3Me3K4: Histone 3 trimethylation of lysine 4
UTR: Untranslated region.
